# Macrophage-Mediated Tissue Vascularization: Similarities and Differences Between Cornea and Skin

**DOI:** 10.3389/fimmu.2021.667830

**Published:** 2021-04-07

**Authors:** Karina Hadrian, Sebastian Willenborg, Felix Bock, Claus Cursiefen, Sabine A. Eming, Deniz Hos

**Affiliations:** ^1^ Department of Ophthalmology, University of Cologne, Faculty of Medicine and University Hospital Cologne, Cologne, Germany; ^2^ Department of Dermatology, University of Cologne, Cologne, Germany; ^3^ Center for Molecular Medicine Cologne (CMMC), University of Cologne, Cologne, Germany; ^4^ Cologne Excellence Cluster on Cellular Stress Responses in Aging-Associated Diseases (CECAD), University of Cologne, Cologne, Germany; ^5^ Developmental Biology Unit, Institute of Zoology, University of Cologne, Cologne, Germany

**Keywords:** macrophages, monocytes, angiogenesis, cornea, skin, lymphangiogenesis

## Abstract

Macrophages are critical mediators of tissue vascularization both in health and disease. In multiple tissues, macrophages have been identified as important regulators of both blood and lymphatic vessel growth, specifically following tissue injury and in pathological inflammatory responses. In development, macrophages have also been implicated in limiting vascular growth. Hence, macrophages provide an important therapeutic target to modulate tissue vascularization in the clinic. However, the molecular mechanisms how macrophages mediate tissue vascularization are still not entirely resolved. Furthermore, mechanisms might also vary among different tissues. Here we review the role of macrophages in tissue vascularization with a focus on their role in blood and lymphatic vessel formation in the barrier tissues cornea and skin. Comparing mechanisms of macrophage-mediated hem- and lymphangiogenesis in the angiogenically privileged cornea and the physiologically vascularized skin provides an opportunity to highlight similarities but also tissue-specific differences, and to understand how macrophage-mediated hem- and lymphangiogenesis can be exploited for the treatment of disease, including corneal wound healing after injury, graft rejection after corneal transplantation or pathological vascularization of the skin.

## Introduction

Macrophages represent highly plastic cells of the hematopoietic system and are found in all tissues (1). Macrophages exert multiple functions including important roles in tissue development, homeostasis, repair and host defense. Several pieces of evidence indicate a critical role of macrophages as mediators of neovascularization (2–4). Furthermore, macrophages have also been shown to mediate repair of damaged vascular tissue (5). Neovascularization occurs very diversly in different tissues. Here we review the role of macrophages in tissue vascularization with a focus on blood and lymphatic vessel formation in cornea and skin. Furthermore, a recent study showed the mediation of vascular tissue repair by macrophages (5).

The cornea is the outer barrier of the eye and is, under healthy conditions, transparent and the major refractive element in the eye. The cornea belongs to the few immune-privileged tissues of the organism and is, unlike the skin, avascular in its healthy state. After severe injury or chronic inflammation, however, corneal avascularity is abrogated as blood and lymphatic vessels can sprout from the adjacent vascularized tissues into the cornea, leading to reduced visual acuity and undesired immune responses (6, 7). However, on the other side previous studies also reported on beneficial roles of lymphatic vessels in the cornea under certain disease conditions (8–10). Corneal macrophages have been shown to be critical mediators of corneal hem- and lymphangiogenesis (2, 11). Similar to the cornea, the skin is an important barrier tissue protecting the body from harmful insults of the environment. In contrast to the avascular cornea, the skin contains a tight network of blood and lymphatic vessels and also several resident immune cell types, including macrophages, which have been shown to be important mediators of skin vascularization. Comparing angiogenesis in both tissues provides an opportunity to highlight molecular principles of macrophage-mediated tissue vascularization.

## Monocyte and Macrophage Heterogeneity

### Origin and Development of Monocytes and Macrophages

Until recently, it was thought that macrophages exclusively originate from hematopoietic stem cell (HSC)-derived monocytes in the bone marrow and are released into the peripheral blood circulation (12). The egression of monocytes from bone marrow into blood requires the expression of the C-C chemokine receptor type 2 (CCR2) (13, 14). Blood monocytes can be subdivided into two subtypes: classical and non-classical monocytes. Classical monocytes are circulating for several days in the blood before leaving the circulation by diapedesis and entering tissues in steady state to replenish the tissue macrophage populations under inflammatory conditions. Non-classical monocytes predominantly remain in the circulation (13) and engage in long-term migration along the endothelium with or against the flow, a process termed patrolling (14). These monocyte subpopulations are reviewed in detail in section 2.2, including their various expression of surface markers.

However, besides macrophages originating and renewing from HSCs, some macrophages develop in the early embryo before the development of HSCs. These cells are termed erythromyeloid progenitors (EMP) (15). It was shown in mice, that macrophages develop in the yolk sac beginning from embryonic day 8.5 (E8.5) (16, 17). Further, the transcription factor myeloblastosis (Myb) is required for the development of HSCs in the bone marrow as well as for the development of all CD11b^high^ monocytes and CD11b^high^ macrophages. For the development of yolk sac-derived F4/80^+^ macrophages in several tissues, including liver Kupffer cells, epidermal Langerhans cells as well as microglia cell populations, Myb was dispensable. In adult mice, these populations can persist independently of HSCs, suggesting that a lineage of tissue macrophages is derived from the yolk sac and is genetically distinct from HSC progeny (18). Additionally, it was shown in mice, that the majority of adult tissue-resident macrophages in various organs like liver, brain, lung and skin originates from an Angiopoietin-1 receptor^+^ (Tie2) cellular pathway generating Colony stimulating factor 1 receptor^+^ (Csf1r) EMPs, which are distinct from HSCs (19, 20). It has been shown that during inflammation there is an expansion of both HSC- and EMP-derived macrophages, presumably performing different functions at different stages of the inflammatory process.

In the cornea, the CCR2^-^ population is already is present in the cornea at E12.5, which are similar to yolk sac-derived macrophages, whereas the CCR2^+^ population does not appear in the cornea until E17.5. Besides the different phenotype and gene expression profile, the role of these populations in corneal wound healing is different. Whereas CCR2^+^ macrophages seem to act pro-inflammatory in an early stage of corneal wound healing, CCR2^-^ macrophages seem to act anti-inflammatory during the later stage of wound healing (21).

### Subpopulations of Murine Monocytes and Macrophages

In mice, the antigenic differentiation of two monocyte subsets was first achieved after the observation that monocytes could be subdivided according to their expression of CCR2, L-selectin (CD62L) and CX3C chemokine receptor 1 (CX3CR1) (13, 22–25). CCR2^+^CD62L^+^CX3CR1^low^ expressing monocytes have pro-inflammatory characteristics and are recruited into the tissue during inflammation, e.g., for host defense, whereas CCR2^-^CD62L^-^CX3CR1^high^ monocytes/macrophages have anti-inflammatory properties and replenish the tissue resident macrophage population, mediate wound healing and patrol the vasculature (13, 26–28). CCR2^+^CD62L^+^CX3CR1^low^ expressing monocytes are known to be the “inflammatory” subset, whereas CCR2^-^CX3CR1^high^ expressing monocytes are considered as the “resident” subset. Besides CCR2 as a marker for monocytes/macrophages, Geissmann et al. identified an additional marker, lymphocyte antigen 6C (Ly6C), for CCR2^+^ monocytes/macrophages (13). In particular, CCR2^+^CD62L^+^CX3CR1^low^Ly6C^+^ monocytes seem to have an outstanding importance for the infiltration into inflamed tissues (29). These cells produce pro-inflammatory cytokines and chemokines, including tumor necrosis factor (TNF)-α, Interleukin (IL)-1β, IL-6, IL-12, IL-23, and C-C motif chemokine 11 (CCL11) (30, 31). Additionally, cells express high levels of Triggering receptor expressed on myeloid cells 1 (TREM1), which can potently amplify pro-inflammatory responses (32).

Fate-mapping studies as well as single cell analyses were recently able to provide major insights into the heterogeneity of macrophages. In this context, a study performed by Yona et al. demonstrated, that tissue resident macrophage populations, including peritoneal, splenic and lung macrophages, as well as liver Kupffer cells, are established prior to birth and are disconnected from monocyte input in adult steady state (27). Furthermore, this study demonstrated that in steady state monocytes with a CX3CR1^int^Ly6C^+^ expression form a short-lived obligatory precursor intermediate for the generation of Ly6C^-^ monocytes, which dynamically control the lifespan of their progenitors (27). A very recent study from Wieghofer et al., provided further insight into the heterogeneity of macrophages in various tissues of the eye, including the cornea (33). In this study, a combination of three techniques, single-cell RNA sequencing, embryonic and adult cell fate mapping and parabiosis with the use of reporter mouse lines was deployed to compare the transcriptional profiles, origin and turnover characteristics of retinal microglia, and resident macrophages in the ciliary body as well as in the cornea (33). Out of 17 different clusters containing CD45^+^CD3^-^CD19^-^Ly6G^-^ cells, five clusters were significantly enriched in the cornea. Furthermore, this study showed, that all investigated compartments of the adult murine eye contained macrophages of prenatal origin that derive either from the yolk sac and/or the fetal liver to various degrees. However, in the cornea of adult mice, macrophages are continuously replaced with cells derived from the definitive hematopoiesis with a short turnover (33).

A number of markers that are expressed by macrophages can be used for characterization and localization during experimental set ups, including F4/80, CD11b and CD68 (34–36). Besides macrophages, F4/80 is also a marker for microglial cells (37). Additionally, myeloid dendritic cells, blood monocytes and eosinophilic granulocytes also express low levels of F4/80 (38), whereas CD11b is also expressed on neutrophils, peritoneal B1 cells, CD8^+^ dendritic cells (DCs), natural killer cells (NK) and a subset of CD8^+^ T cells (39–41). CD11b is also highly expressed in CD4^+^ conventional DCs and in conventional DCs type 2 regardless of their CD4 expression (42). CD68 is also present on basophils, dendritic cells, fibroblasts, Langerhans cells, mast cells, CD34^+^ progenitor cells, neutrophils, osteoclasts, activated platelets and B and T cells (43, 44). Additional markers widely used for the characterization of tissue resident macrophages are CD64 and Mer tyrosine kinase (MerTK). A combination of different markers including CD11b, F4/80, CD64, MerTK and CD68 for flow cytometry is commonly used to characterize tissue-resident macrophages (45). Furthermore, the characterization of macrophages is also possible using immunofluorescence. For example, Saylor et al. developed an automated, multiplexed staining approach including anti-CD68, -CD163, -CD206, -CD11b, and -CD11c antibodies to identify macrophages in tumor tissue (46).

Interestingly, macrophages share the marker Lymphatic Vascular Endothelial Hyaluronan Receptor 1 (LYVE-1) with lymphatic endothelial cells (LECs) (47). Therefore, these cell types also have to be discriminated, e.g. by using further specific markers for LECs such as the transcription factor Prospero homeobox protein (Prox-1) (48) and the membrane glycoprotein Podoplanin (6, 49, 50). Besides lymphatic vascular endothelium, LYVE-1 serves for both, macrophages and LECs as receptor during hyaluronate metabolism and angiogenesis (2, 3, 6, 51–53). Intriguingly, a recent study of Chakarov et al. has shown that two independent monocyte-derived tissue resident macrophage populations exist across various tissues with specific niche-dependent phenotypes and functional programming, distinguished by their LYVE-1, MHC II and CX3CR1 expression pattern (54). LYVE-1^low^MHC II^high^CX3CR1^high^ macrophages are preferentially located, but conserved, in sub tissular niches located adjacent to nerve fibers, whereas LYVE-1^high^MHC II^low^CX3CR1^low^ macrophages are preferentially located adjacent to blood vessels (54). LYVE-1^low^MHC II^high^CX3CR1^high^ macrophages exhibit potent immune-regulatory potential, while LYVE-1^high^MHC II^low^CX3CR1^low^ macrophages are able to express higher levels of genes which are involved in wound healing, repair, and fibrosis, as well as blood vessel morphology and leukocyte migration (54).

### Activation Phenotypes of Macrophages

A plethora of functional macrophage phenotypes exist. For a long time, macrophage phenotypes were classified into two polarized macrophage subtypes, depending on their activation state. The “classical” activation of macrophages occurs *via* stimulation by pro-inflammatory mediators, e.g. Interferon (IFN)-*γ*, TNF-α or lipopolysaccharides (LPS). These macrophages show an increased expression of pro-inflammatory cytokines such as TNF-α, IL-6 and IL-12, increased antigen presentation and production of nitrogen and oxygen radicals as well as increased microbicidal activity (55). This macrophage phenotype occurs primarily in early phases of inflammatory responses. In contrast, “alternative” activation of macrophages is mediated by the Type 2 cytokines IL-4 and IL-13 which induce the expression of hallmark genes such as *Retnla* (resistin-like molecule alpha), *Chil3* (chitinase-like 3), and *Arg1* (arginase 1) (55, 56). IL-4/IL-13-activated macrophages show an increased activity in signaling pathways that are important for the termination of an immune response, leading to an increase of the expression of prophagocytic, antioxidant and motility-enhancing factors, while the expression of pro-inflammatory factors is decreased (55). Furthermore, IL-4Rα-activated macrophages are crucially involved in tissue repair, evidenced by defective skin wound healing in *Il4ra* ^fl/−^ *Lyz2-cre* mice (57). In this study, IL-4Rα-activated macrophages were shown to have a major impact on the collagen-modifying function of fibroblasts and thereby on scar formation (57). Macrophages in *Il4ra* ^fl/−^ *Lyz2-cre* mice fail to initiate an essential repair program rather than an unrestrained pro-inflammatory response which was reported in prior studies by Chen et al. (58). It is clear, however, that this subdivision is an oversimplification and only reflects two extremes of polarization and that in tissues a wide range of activation states exist in parallel (56).

## Corneal Macrophages

Previous studies have demonstrated that resident tissue macrophages and antigen-presenting cells (APCs) are present in various tissues of the eye, including the iris, ciliary body, uvea, retina, conjunctiva, and cornea (59–63). It was also shown that macrophages express low levels of MHC II and further costimulatory molecules, which enables them to act as APCs, although working less efficiently than dendritic cells due to their relatively reduced ability to migrate and prime naïve T cells (42, 64–67). In this regard, it should be noted that there are several lines of evidence that Langerhans cells are not DCs but rather a population of specialized macrophages (42, 68). Furthermore, it was demonstrated that a high number of CD45^+^ cells (leucocytes) with pleomorphic and dendriform morphology were found within the pericentral and central region of the corneal stroma (69). It was demonstrated, that all CD45^+^ cells in the corneal stroma are also CD11b^+^ and around 50% of the CD45^+^ cells were also F4/80^+^. Approximately 30% of all CD45^+^ cells and 50% of F4/80^+^ cells co-expressed MHC II, whereas only a very small number of the CD45^+^ cells were positive for CD11c (dendritic cells) or Ly6G (granulocytes) (69). In short, this study shows that two different subsets of F4/80^+^ macrophages exist in the cornea, discriminated based on their MHC II expression. No T cells and NK cell markers were found in the naïve corneal stroma, indicating that all cells identified in the stroma were of the myeloid lineage (69).

Early experiments from Streilein and colleagues indicated that the cornea has no MHC II^+^ cells capable of stimulating acute allogeneic rejection (70). Subsequently, two independent studies described a network of CD11b^+^ macrophage-like cells as well as a significant number of CD45^+^ leukocytes in the stroma and CD11c^+^ DCs in the corneal epithelium of normal mouse corneas (69, 71). MHC II^+^ cells were typically located in the periphery of the corneal epithelium with a dendritic morphology in various species, including mice (72) and humans (72–74). It was also shown, that CCR2^-^ macrophages, which already exist in the cornea at E12.5, may be derived from progenitors originating in the fetal liver or earlier yolk sac (17), as yolk sac progenitors seem to express only low levels of CCR2. The CCR2^+^ population does not appear in the cornea until E17.5 (21). It was also demonstrated that CCR2^−^ macrophages in the cornea were mainly maintained through local proliferation and were rarely replaced by blood monocytes, whereas CCR2^+^ macrophages with a lower ability to proliferate were replaced by blood monocytes (21). It was proposed that the turnover rate of bone marrow-derived CX3CR1^+^ cells in the cornea is fast (approximately 40% in 4 weeks) compared to other non-lymphoid tissues (75), including lung, liver and brain [turnover of less than 5% in 4 weeks (76)]. This study also proposed, that the higher turnover rate in the peripheral cornea is a reflection of the close location to the vascular limbus (75).

Taken together, macrophages are also present in the immune-privileged cornea, preferable in the periphery of the corneal stroma. However, macrophages are also found occasionally in the central cornea, with a possible origin in the bone marrow as well as in the yolk sac. Nonetheless, the normal central cornea is devoid of MHC II^+^ cells (70, 77).

## Corneal Hem- and Lymphangiogenic Privilege

**Figure 1:**
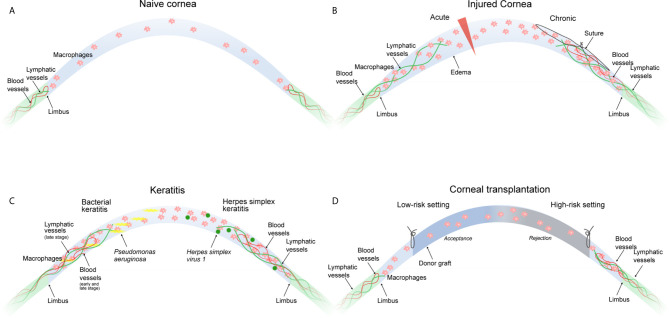
Macrophages in corneal neovascular disease. **(A)** The naive cornea is devoid of blood and lymphatic vessels. Few immune cells including macrophages are present in the peripheral cornea and to a lesser extent in the central cornea. **(B)** An acute incisional injury (left) leads to the ingrowth of lymphatic, but not blood vessels. A chronic injury (suture placement, right) leads to the ingrowth of blood and lymphatic vessels. Corneal neovascularization in both injury models critically depends on the presence of corneal macrophages. **(C)** Bacterial keratitis (left) leads to ingrowth of blood vessels in the early and late stage of infection, whereas lymphatic vessels only appear in the late stage of infection. Both are critically dependent on the presence of corneal macrophages. HSV-1 keratitis (right) leads to the ingrowth of blood and lymphatic vessels independently of the presence of corneal macrophages. **(D)** The low-risk corneal transplantation setting (left) without pre-existent corneal neovascularization usually does not result in graft rejection, whereas the high-risk corneal transplantation setting (right) with pre-existent blood and lymphatic vessels is likely to result in graft rejection. Macrophage depletion significantly improves graft survival. All settings **(B–D)** result in the accumulation of corneal macrophages.

In most tissues and organs, blood and lymphatic vascular systems are essential to supply organs and tissues with oxygen and nutrients, to drain redundant fluid and metabolites and to support the immune system to protect the body against foreign organisms (78, 79). However, there are some tissues that do not rely on the presence of blood and/or lymphatic vessels to maintain their unique structure and fulfill their function. The cornea is one of these rare tissues that actively maintains an avascular state, which is called “corneal (lymph)angiogenic privilege” (80) (**Figure 1A**). In general, it seems that the maintenance of corneal avascularity does not only occur as a result of the upregulation of anti-angiogenic factors, but also from the downregulation of pro-angiogenic factors in the healthy cornea (81). It has been shown in this context, that the balance between angiogenic and anti-angiogenic factors especially in the corneal epithelium plays an important role in corneal avascularity (82–84). This includes the pro-angiogenic factors fibroblast growth factor-2 (FGF-2), vascular endothelial growth factor (VEGF) and the transforming growth factor-α (TGF-α) (85, 86), as well as the anti-angiogenic factors including endostain (87), thyrosinase (88), semaphorin 3F (89), angiostatin (90) and thrombospondin (TSP-1) (91–96). TSP-1, an anti-angiogenic, multifunctional extracellular matrix protein, plays an interesting role in the lymphangiogenic privilege of the cornea. It was shown that aged (6-month-old) TSP 1^-/-^ mice develop a spontaneous ingrowth of lymphatic vessels into the cornea, which was also shown in mice lacking the TSP-1 receptor CD36 (95). Mechanistically, it was demonstrated that TSP-1 down-regulates the expression of VEGF-C *via* CD36 in macrophages, proposing that macrophages are involved in the maintenance of the lymphangiogenic privilege of the cornea (95). Additionally, factors which can act pro- as well as anti-angiogenic are also present in the cornea, including TGF-β (97–99). It was further demonstrated that corneal avascularity is dependent on the expression of soluble VEGF receptor 1 (sVEGFR-1) in the corneal epithelium (100). The lack of sVEGFR-1, which serves as an endogenous VEGF−A trap (101), abolishes corneal avascularity in mice (100). A further crucial regulator of lymphatic vessel growth is sVEGFR-2, which inhibits lymphangiogenesis by blocking VEGF−C function (102). Further studies showed that also sVEGFR-3 is expressed in the cornea and is essential for corneal alymphaticity (103). This protein binds and sequesters VEGF-C, thereby blocking signaling through VEGFR-3 and suppressing lymphangiogenesis induced by VEGF-C. The knockdown of sVEGFR-3 leads to neovascularization in the mouse cornea. In contrast, the overexpression of sVEGFR-3 inhibits neovascularization in a murine suture injury model (103). Membrane-bound VEGFR-3 is also strongly constitutively expressed by the corneal epithelium and is mechanistically responsible for suppressing inflammatory corneal hem- and lymphangiogenesis (104).

Additionally, the corneal (lymph)angiogenic privilege is provided by the special anatomy of the cornea, which ensures a constant dehydration, resulting in periodically ordered, tightly packed collagen lamellae and a compact keratocyte network. The periodicity is highly dependent on the state of stromal hydration. In case of stromal edema, the compactness of the stroma is disturbed and therefore vessels can easier grow in-between the lamellae (105, 106). However, in early studies it was shown, that corneal swelling can occur without vascularization (107). Currently, it is thought that also the limbus may act as a physical and physiological barrier to invading vessels in the immediate vicinity and might also prevent an overgrowth of the cornea with conjunctival epithelial cells (108–112). Disturbances of limbal stem cells for example by UV-light may also deregulate the (lymph)angiogenic privilege of the cornea (113). However, other studies question the concept of the limbal barrier to corneal vascularization as the basis of corneal angiogenic privilege (81, 114).

Besides being a (lymph)angiogenic privileged tissue, it is well established that the cornea is also an immune-privileged tissue (115). Anterior chamber-associated immune deviation (ACAID) is an example for this immune privilege, which partially depends on an eye-derived, suppressor-inducing macrophage subset that acts through NK cells (116, 117) and suppresses antigen-specific, delayed-type hypersensitivity (DTH) (118). It was shown that corneal immune privilege is co-responsible for the (lymph)angiogenic privilege of the cornea (119). Interestingly, several molecules are involved in maintaining both corneal angiogenic and immune privilege such as the thrombospondins (95, 96).

Due to the here described (lymph)angiogenic privilege, corneal wound healing after (minor) injury usually takes place without any neovascularization. However, after severe injury, e.g. as a result of trauma, infection, and inflammatory or degenerative disorders, the (lymph) angiogenic privilege of the cornea might be overwhelmed (“threshold concept”), leading to the invasion of blood and/or lymphatic vessels into the cornea (corneal neovascularization).

## Corneal Neovascularization in Pathology and Disease

### Breakdown of Corneal (Lymph) Angiogenic Privilege

Corneal avascularity is highly important for the maintenance of corneal transparency, ensuring the basis of good visual acuity. However, a variety of diseases and surgical manipulations can lead to the breakdown of the hem- and lymphangiogenic privilege of the cornea resulting in pathological corneal hem- and lymphangiogenesis. Diseases that can be associated with corneal neovascularization include inflammatory disorders, corneal graft rejection after transplantation, infectious keratitis, contact lens-related hypoxia, alkali burns, stromal ulceration, or limbal stem cell deficiency. In these conditions, the balance between pro-angiogenic and anti-angiogenic factors is disturbed and leads to an upregulation of pro-angiogenic factors, and a downregulation of anti-angiogenic factors followed by neovascularization (120–122). Blood vessels directly reduce corneal transparency if growing into the optical zone or due to secondary effects such as hemorrhage and lipid exudation through immature and leaky capillaries. Unlike blood vessels, clinically invisible lymphatic vessels do not reduce the transparency of the cornea. However, they contribute to various inflammatory diseases of the ocular surface, including corneal transplant rejection, dry eye disease (DED) and ocular allergy (7, 123). In those diseases, the corneal lymphatic vessels facilitate the migration of APC from the ocular surface to the regional lymph nodes, which induces undesired immune responses (124–126). On the other hand, lymphatics may also be involved in draining excess tissue fluid thus contributing to corneal transparency and vision (9).

### Role of Macrophages in Corneal Neovascularization

Macrophages play a pivotal role in corneal neovascularization. Macrophages are able to secrete paracrine factors, such as VEGF-A, which promotes hem- and lymphangiogenesis by binding on VEGFR-2 (2), and VEGF-C and VEGF-D, which promote lymphangiogenesis by binding to VEGFR-3 (127, 128). In addition, macrophages also express VEGFR-1 and VEGFR-3 which both may mediate chemotactic effects in myeloid cells and thereby perpetuate an inflammatory hem- and lymphangiogenic response (“immune amplification”) (2). Notably, so far it is not reported in the literature whether macrophage-derived VEGF-A is critical for corneal vascularization, and it is unclear by which other mediator macrophages precisely might mediate corneal vascularization. Besides secreting lymphangiogenic and angiogenic growth factors (2), macrophages also directly contribute to corneal lymph vessel formation by integrating into newly formed corneal lymphatic vessels (3). That means macrophages have a dual important role in mediating corneal lymphangiogenesis (129). Furthermore, macrophages seem to be essential also for maintenance of (corneal) lymphatics (130). It was shown that depletion of macrophages significantly reduces corneal hem- and lymphangiogenesis (2, 131, 132). Recent studies have also identified distinct functions of early- versus late-phase corneal wound macrophages in hem- and lymphangiogenesis: whereas early-phase wound macrophages are essential for the initiation and progression of injury-mediated corneal hem- and lymphangiogenesis, late-phase wound macrophages control the maintenance of established corneal lymphatic vessels, but not blood vessels (10, 11). Furthermore, studies indicate that the type of corneal damage controls the hem- and lymphangiogenic potential of corneal macrophages: whereas an acute perforating incision injury induced wound macrophages with lymphangiogenic, but not hemangiogenic potential with an increased expression of VEGF-C and D, suture placement into the corneal stroma provoked wound macrophages with hem- and lymphangiogenic potential (**Figure 1B**). Interestingly, in a model of *Pseudomonas aeruginosa*-induced bacterial keratitis, corneal hemangiogenesis was induced in early as well as late stages, whereas lymphangiogenesis was induced solely in late stages, which was strongly dependent on corneal macrophages (**Figure 1C**) (10). Taken together, the hem- and lymphangiogenic potential of corneal wound macrophages is determined by the type of the corneal damage and the phase of corneal injury (11). However, based on currently available data, it seems not possible to speculate or conclude whether different macrophage populations or different activation phenotypes are separately regulating corneal hem- and/or lymphangiogenesis.

Another possibility of macrophages promoting hemangiogenesis is VEGF-independent, as macrophages might act as bridge cells on the tips of sprouting lymphatics guiding the cells into finding and anastomosing with tip cells from other sprouting lymphatics (133, 134). However, this pathway has not been shown for corneal neovascularization so far.

It should be noted that corneal neovascularization might also occur independent of macrophages. An example is corneal lymphangiogenesis induced by Herpes simplex virus 1 (HSV-1) (135). It was shown in this context that lymphangiogenesis depends on VEGF-A/VEGFR-2 signaling but not on VEGFR-3 ligands. Importantly, macrophages were not the source of VEGF-A and did not play a role in the induction of the lymphangiogenic response. Infected epithelial cells were discovered as the primary source of VEGF-A in this model, suggesting that HSV-1 directly induces vascularization of the cornea through up-regulation of epithelial VEGF-A expression and not *via* macrophages (**Figure 1C**).

We have recently demonstrated an important role of Interleukin-10 (IL-10)-activated macrophages in inflammatory corneal neovascularization. In particular, we could show that the multifunctional cytokine IL-10, which acts anti-inflammatory as well as immune-regulatory, controls the corneal lymphangiogenesis and the resolution of corneal inflammation *via* macrophages (8). In healthy corneas, the expression of IL-10 was unverifiable, however macrophages which infiltrated inflamed corneas after corneal injury showed a strong increase in IL-10 expression. *In vitro* stimulation of macrophages with IL-10 led to an anti-inflammatory, but surprisingly pro-lymphangiogenic phenotype, characterized by an upregulation of VEGF-C. In IL-10 deficient mice, corneal injury resulted in both reduced expression of VEGF-C and reduced corneal lymphangiogenesis. However, the loss of IL-10 had no effect on corneal hemangiogenesis (8). The deletion of the central mediator of IL-10 signaling, Signal transducer and activator of transcription 3 (Stat3), specifically in myeloid cells resulted in reduced corneal lymphangiogenesis and persistent corneal inflammation in injured corneas, reinforcing the critical role of IL-10^+^ macrophages in the regulation of corneal lymphangiogenesis and inflammation (8). These findings indicate that IL-10 leads to an anti-inflammatory but pro-lymphangiogenic VEGF-C secreting macrophage phenotype during an inflammatory corneal response. These macrophages can induce the activation and growth of lymphatic vessels, leading to an egress of inflammatory cells and the termination of the local inflammatory response (8).

#### Role of Specific Macrophage Subpopulations in Corneal Neovascularization

Not much is known about the role of specific macrophage subpopulations during corneal neovascularization. In a mouse model with either a knockout of CCR2 or CX3CR1, or a macrophage depletion model, corneal neovascularization was induced by alkali injury (136). It was shown in this model that CCR2-deficient mice exhibited reduced corneal neovascularization with reduced macrophage infiltration, whereas corneal neovascularization in CX3CR1-deficient mice was increased with reduced macrophage infiltration. Macrophage depletion did not affect corneal neovascularization, which is in contrast to other studies showing that macrophage depletion inhibits corneal neovascularization (2, 11). It should also be noted that this study only assessed corneal hemangiogenesis and did not analyze corneal lymphangiogenesis (136).

#### Corneal Transplantation and the Role of Macrophages in Transplant Rejection

Corneal transplantation (keratoplasty) is the most frequently performed form of transplantation worldwide, with more than 100.000 transplants per year (137). Due to the immunological privilege of the cornea, keratoplasty usually results in good transplantation outcomes (138, 139).

This immunological privilege of the cornea actively suppresses immune responses against the allograft, enabling the transplantation of HLA-mismatched corneal grafts without the need of systemic immunosuppression (119, 140). However, the immunological privilege of the cornea is not invulnerable. In this regard, it has been shown that e.g. severe inflammation can overcome the immunosuppressive mechanisms of the cornea and results in an immunological scenario similar to solid organ transplantation, where e.g. HLA-matching and systemic immunosuppression are necessary to avoid immune-mediated allograft rejection (140–142). Thus, in eyes with compromised immunological privilege graft failure caused by immune rejection continues to be a major barrier to transplantation success. Keratoplasty can thus be divided into two risk categories dependent on the immunological status of the host cornea. In the so-called low-risk setting, the immunological privilege of the cornea is intact and graft rejection is unlikely. In contrast, in the high-risk setting, the immunological privilege of the cornea is lost, and the risk of graft rejection is significantly increased (**Figure 1D**) (141, 143). It is now widely accepted that the vascularization status of the cornea is the most important factor defining the low- or high-risk status of the host. Avascular hosts are generally considered as low-risk hosts, whereas vascularized hosts are generally considered as high-risk hosts. In this regard, it has been demonstrated that preexistent pathological lymphatic vessels facilitate trafficking of APCs from the graft site to regional draining lymphoid tissues where APCs can then present alloantigens to host T cells. In fact, in the murine model of corneal transplantation it was clearly shown that lymphatic and not blood vessels determine the high-risk state of neovascularized recipient beds (124). Pathological preexistent blood vessels then facilitate the homing of primed effector T cells to the graft site where allorejection is mediated (141).

Early research in rat eyes have demonstrated reduced rejection rates of corneal transplants after depletion of macrophages, demonstrating the crucial role of macrophages in corneal transplantation (131, 144). In this study, all transplants in the control group were rejected within 17 days, whereas the transplants in macrophage-depleted eyes were not rejected during the entire follow-up period over 100 days post transplantation. Additionally, a reduced vascular response in clodronate-treated recipient corneas was observed, indicating that a positive graft outcome might (indirectly) depend on corneal vessels (145). In a pre-vascularized high-risk transplantation model, depletion of macrophages did not fully prevent, but significantly delayed graft rejection (146). Additionally, corneal neovascularization was significantly reduced after macrophage depletion in this model (131). It was also shown that the depletion of macrophages results in a strongly downregulated local and systemic immune response after transplantation (144). Additionally, large numbers of CD11b^+^, F4/80^+^ and iNOS^+^ (inducible nitrous oxide synthase) macrophages infiltrated corneal allografts during rejection in mice, indicating that these cells might directly contribute to corneal graft rejection (147). As the use of clodronate liposomes to deplete macrophages may also affect APCs like DCs (148, 149), the conclusions regarding the role of macrophages indicated by these experiments have to be drawn with caution, because the findings may also be indirectly caused by the affection of other cell types.

The gold standard to prevent or treat corneal graft rejection is the application of glucocorticosteroids (150, 151). The treatment with glucocorticosteroids leads to decreased corneal infiltration of macrophages and reduced expression of pro-inflammatory cytokines, such as TNF-α and IL-1β (152). Furthermore, glucocorticosteroids also significantly reduce progressive corneal hem- and lymphangiogenesis, which likely contributes to reduced rejection rates (152). A recent study showed that the topical application of VEGF-C and VEGF-C prevents the in growth of lymphatic vessels into the murine cornea after suture-placement in a high-risk corneal transplantation model (153). Further it was shown that the topical application of VEGF-C and VEGF-D increases the number of macrophages, together with a decreased expression of the anti-inflammatory macrophage marker Arginase-1, as well as of the immune modulatory cytokine TGF-β (153). Moreover, it was shown, that corneal crosslinking with UVA light together with riboflavin leads to a regression of preexisting blood and lymphatic vessels significantly *via* induction of apoptosis in vascular endothelial cells with a reduced number of macrophages and CD45^+^ cells (154).

In summary, modulation of corneal macrophages does not only alter the inflammatory and cellular milieu in transplanted corneas, but also affects corneal neovascularization (131). Thus, the beneficial effect of macrophage depletion on graft survival may be attributable to diminished corneal neovascularization. The effect of a modulation of macrophage function without altering the corneal vascular response in the context of transplantation was not shown yet.

#### Novel Beneficial Functions of Macrophage-Mediated Corneal Lymphangiogenesis in the Regulation of Corneal Edema and Transparency

Outside the eye, e.g. in the skin, it is well-established that lymphatic vessels regulate tissue pressure, allow fluid drainage and prevent the development of edema (155). However, so far it remained elusive, whether lymphatic vessels have similar functions in the cornea and are involved in the regulation of edema and transparency. Recently, we have therefore investigated whether an incisional corneal injury that leads to acute corneal edema and transparency loss is accompanied by the ingrowth of lymphatic vessels into the cornea and whether corneal lymphangiogenesis potentially contributes to the healing response. This type of corneal injury indeed resulted in a transient ingrowth of lymphatic vessels into the cornea (9). Importantly, blockade of lymphangiogenesis resulted in increased corneal thickness, arguably due to delayed drainage of corneal edema, and a trend towards prolonged corneal opacification (9). Corneal lymphangiogenesis after this type of corneal injury was dependent on the presence of macrophages, as macrophage depletion using clodronate liposomes significantly reduced corneal lymphangiogenesis (11). This study indicates that corneal lymphangiogenesis plays an important role in the regulation of corneal edema and transparency, and is also in line with the finding that corneal lymphangiogenesis may be beneficial in bacterial keratitis by improving corneal edema in later disease stages (10). Whether this holds true also for chronic forms of (mild) corneal edema needs to be studied. That would open completely new therapeutic options for common diseases leading to corneal transplantation.

## Similarities and Differences of Macrophage-Mediated Neovascularization in Cornea and Skin

Both cornea and skin are protective barrier organs that shield the body from harmful insults of the environment. The skin consists of the epidermis, the dermis and the dermal white adipose tissue. In contrast to the avascular cornea, skin contains in the steady state an interwoven network of blood and lymphatic vessels, in which diverse leukocyte subsets such as dermal dendritic cells, T cells, and macrophages are embedded (156). Studies in *PU.1^-/-^* mice, in which the myeloid cell lineage is severely impaired and which lack skin resident F4/80^+^ myeloid cells, revealed that macrophages are dispensable in developing skin vasculature (157, 158). However, macrophages critically regulate pericyte development in the skin and the dermal lymphatic vessel caliber, as shown in both, macrophage deficient *PU.1^-/-^* and *Csf1r^-/-^* mice (158, 159)

Based on their spatial relationship to dermal vessels, skin-resident macrophages were defined as perivascular (direct contact with vessel or < 15 µm from vessel) or interstitial macrophages (> 15 µm from vessel) (156, 160). In human skin the proportion of perivascular macrophages (PVMs) increases from the apical towards the deep dermis (156). PVMs are considered to have maintenance functions in steady state tissues, such as regulating vascular permeability and scavenging blood-derived pathogens (160). Under inflammatory conditions PVMs have been shown to guide neutrophils during extravasation into infected dermis and to regulate dendritic cell clustering in perivascular areas (161, 162). Interestingly, a subset of skin PVMs has been identified, which protrudes across endothelial junctions into microvessels in order to take up macromolecules from the blood stream (163).

Comparable to the inflamed cornea, macrophages have a critical function in regulating neovascularization in skin under inflammatory conditions. Vascularization upon skin injury and during the wound healing response is a useful experimental model to study the molecular basis of neovascularization (164). Upon skin injury a high number of myeloid cells is recruited from the blood and forms together with mainly fibroblasts, myofibroblasts and endothelial cells a highly vascularized granulation tissue within several days (165). In this model, angiogenesis is at the core of an efficient repair response and critical for timely wound closure. With time, inflammation declines, and the granulation tissue matures into scar tissue, characterized by regression of blood and lymph vessels (29, 166). By using mouse models of diphtheria toxin-inducible cell depletion, several groups provided evidence that angiogenesis in the developing granulation tissue requires myeloid cells (167–169). Similar to the cornea, early-phase wound macrophages in skin wounds were shown to be essential for the initiation of wound vascularization (11, 167). Specifically, myeloid cell–derived VEGF−A was shown to be critical for the induction of wound angiogenesis and tissue growth during the early phase of skin repair (29). While the role of specific macrophage populations in corneal neovascularization is not entirely resolved, blood-derived inflammatory CCR2^+^Ly6C^high^ monocytes/macrophages were identified as the critical source of VEGF−A in skin wounds (29).

### The Role of HIF During Macrophage-Mediated Vascularization in Skin and Cornea

A major transcription factor, which is stabilized during physiological skin wound healing and which is required for the induction of *Vegfa*, is Hypoxia-Inducible Factor 1 α (HIF-1α) (170, 171). Stabilization of HIF-1α is impaired in a diabetic environment and in aged mice, conditions with a typically impaired wound healing response. Interestingly, angiogenesis and wound closure are improved in diabetic mice when HIF-1α is stabilized (170–174). Up to date, in classic repair models in the cornea (e.g. corneal incision injury, suture-induced corneal neovascularization) a functional impact of HIF-1α stabilization on neovascularization and repair has not been described. Of note, in a mouse model of corneal HSV−1 infection, hypoxia in the cornea and subsequent stabilization of HIF-1α in immune cells has been shown (175). However, whether HIF-1α is activated specifically in macrophages and whether this has a functional impact in this infection model, remains open. Chen et al. could show an inhibition of VEGF expression and corneal neovascularization by shRNA targeting HIF-1α in a mouse model of closed eye contact lens wear (176). In skin, wound healing studies using mouse models with cell type-specific *Hif1a* gene deletion revealed that endothelial cell-, fibroblast-, and epidermis-specific HIF-1α are critical for *Vegfa* expression, angiogenesis, and timely wound closure (177–179). However, direct evidence that myeloid cell-derived *Vegfa* expression depends on HIF-1α activation in early phase wound macrophages is lacking. Yet, the critical role of HIF-1α in regulating *Vegfa* expression in macrophages and the inflammatory phenotype of skin macrophages is well documented (180), proposing that HIF-1α might regulate *Vegfa* expression in early phase wound macrophages. Both, hypoxia and inflammatory stimuli such as TNF-α, IL-1β, and bacterial products have been shown to stabilize HIF-1α in a NF-κB (nuclear factor kappa-light-chain-enhancer of activated B cells)-dependent manner (181, 182). Interestingly, by day 1 after injury the wound tissue was shown to be normoxic, while macrophages already expressed *Vegfa* (183), indicating that in the very early phase of healing other signals than hypoxia might induce an angiogenic phenotype in macrophages. Recently, mitochondrial metabolism has been identified as critical regulator of HIF-1α activation in macrophages *in vitro*. Under inflammatory conditions, macrophages repurpose their mitochondria from ATP production towards production of mitochondrial reactive oxygen species (mtROS), which stabilize HIF-1α independently of hypoxia (184). In future studies it will be interesting to understand whether mtROS operate in wound macrophages to mediate the wound angiogenic response.

### Macrophage-Mediated Lymphangiogenesis in the Skin

As revealed by imaging of lymphatic vessels in Vegfr3 reporter mice (*Vegfr3* ^EGFPLuc^), lymphangiogenesis is a transient process during skin wound healing, peaking in the mid-phase of healing and returning back to basal levels after re-epithelialization is completed (166). In experimental mouse models of skin wound healing and contact hypersensitivity, treatment with the synthetic glucocorticoid dexamethasone blocks lymphangiogenesis, showing that lymphangiogenesis is tightly connected with the inflammatory response in the skin (166). A similar finding was reported in corneal repair; after suture placement in the cornea corticosteroids were identified as strong inhibitors of corneal lymphangiogenesis (152). Furthermore, by phase-specific macrophage depletion our group has shown that in the injured cornea early-phase macrophages are essential for initiation of lymphangiogenesis (11). Yet, the assessment of lymphangiogenesis in skin wounds upon diphtheria toxin-mediated macrophage ablation is lacking (11, 167–169). However, similar to the inflamed cornea, independent groups identified F4/80^+^/LYVE-1^+^ lymphatic structures in early granulation tissue after excisional punch injury, indicating that macrophages contribute to lymphatic vessels during physiological skin repair (185, 186). The crucial function of macrophages in regulating lymphangiogenesis in skin wounds was further demonstrated by treating wounds of diabetic mice with IL-1β-activated macrophages, which resulted in the formation of granulation tissue and of new F4/80^+^/LYVE-1^+^ lymphatic vessel structures (186). Further, macrophages are well-known sources of lymphangiogenic paracrine factors. The critical impact of macrophage-derived VEGF-A, VEGF-C, and VEGF-D on lymphangiogenesis was shown by Kataru et al. in an ear skin inflammation model (187). Following the intradermal injection of Toll-like receptor (TLR) ligands, depletion of macrophages by clodronate or blockade of VEGF−A or VEGF−C/D resulted in significantly attenuated lymphangiogenesis in the inflamed skin and impaired inflammation resolution (187). Expression of *Vegfc* in skin macrophages has been shown to be controlled by the transcription factor tonicity-responsive enhancer-binding protein (TonEBP, also known as NFAT5) (188). In this study, deletion of *Nfat5* specifically in myeloid cells prevented high salt diet-induced *Vegfc* expression and subsequently lymphangiogenesis in the skin (188). Interestingly, in a mouse model of bacterial skin infection it was found that salt accumulated at the site of the skin lesion drives inflammatory macrophage activation *via* TonEBP to facilitate pathogen removal (189). Whether myeloid cell-specific TonEBP has a function during skin and corneal repair is unknown. It will be interesting to study in the future the interrelationship between salt concentrations, TonEBP activation, lymphangiogenesis, and macrophage activation in both tissues.

## Conclusions and Perspectives

Macrophages originating either from HSCs or the yolk sack, can act inflammatory as well as anti-inflammatory, characterized by their surface marker expression. In the eye, several studies have demonstrated, that resident tissue macrophages as well as APCs are present in most tissues of the eye, including the corneal limbus. Although, the cornea is an immune-privileged tissue, macrophages are also present in the cornea, preferable in the periphery of the corneal stroma. However, macrophages are also found occasionally in the central cornea, with a possible origin in the bone marrow as well as in the yolk sac. Nonetheless, the normal central cornea is devoid of MHC II^+^ cells.

The healthy cornea is one of the rare avascular tissues of the organism, following the “corneal (lymph)angiogenic privilege”, which is actively maintained by the balanced expression of pro- and anti-angiogenic factors. Is this equilibrium somehow disturbed (e.g. in infectious keratitis, stromal ulceration, or limbal stem cell deficiency), the imbalance of these factors leads to a pathological vascularization of the cornea, often mediated by macrophages that secrete paracrine factors, such as VEGF-A promoting hem- and lymphangiogenesis, and VEGF-C and VEGF-D specifically promoting lymphangiogenesis. Additionally, the expression of VEGFR-1 and VEGFR-3 mediates chemotactic effects and perpetuates an inflammatory hem- and lymphangiogenic response. However, not much is known about the role of specific macrophage subpopulations during corneal neovascularization and the available data is still ambiguous. Recently, factors like IL-10 found their way into corneal neovascularization research by acting anti-inflammatory as well as immune-regulatory and by controlling corneal lymphangiogenesis and the resolution of corneal inflammation *via* macrophages. In corneal transplantation, graft survival can be increased by depleting corneal macrophages, which may also be attributable to diminished corneal neovascularization.

Outside the eye, e.g. in the skin, it is well-established that lymphatic vessels regulate tissue pressure, allow fluid drainage and prevent the development of edema. Not much is known about a similar concept in the cornea, but we recently demonstrated increased corneal thickness after blockade of lymphangiogenesis in an acute corneal wound model. If this might also be true for chronic forms of (milder) corneal edema, it would open new therapeutic options for common edematous corneal diseases necessitating corneal transplantation.

In contrast to the avascular cornea, the skin contains in the steady state an interwoven network of blood and lymphatic vessels containing various types of immune cells, including macrophages. Under inflammatory conditions, macrophages have a comparable, critical function in regulating neovascularization in skin and cornea. Similar to the cornea, early-phase wound macrophages in skin wounds were shown to be essential for the initiation of wound vascularization. In contrast to the role of specific macrophage populations in corneal neovascularization, it was shown, that blood-derived inflammatory CCR2^+^Ly6C^high^ monocytes/macrophages act as the critical source of VEGF−A in skin wounds. In contrast to corneal wound healing, in skin HIF-1α is an important transcription factor which is required for the induction of *Vegfa.* However, it should be mentioned, that stabilization of HIF-1α in immune cells has been shown in a mouse model of corneal HSV−1 infection. The use of glucocorticoids blocks lymphangiogenesis, which is similar in the skin and the cornea. Phase-specific macrophage depletion studies have shown that in the injured cornea early-phase macrophages are essential for initiation of lymphangiogenesis. By now, this information for skin is lacking, whereas it is known, that macrophages play a crucial role in lymphangiogenesis during skin repair by the expression of VEGF-C. One transcription factor acting as an inducer for VEGF-C in macrophages is TonEBP. However, whether TonEBP plays also a crucial role during lymphangiogenesis during corneal wound repair is still unknown.

## Author Contributions

Writing—original draft preparation, KH, SW. Writing—review and editing, FB, CC, SE, DH. Funding acquisition, FB, CC, SE, DH. All authors contributed to the article and approved the submitted version.

## Funding

Financial Support: German Research Foundation (DFG): FOR2240 “(Lymph)angiogenesis and Cellular Immunity in Inflammatory Diseases of the Eye”, Cu 47/4-2 (CC), Cu 47/6-1 (CC), Cu 47/9-1 (CC), Cu 47/12-2 (CC), HO 5556/1-2 (DH), EM 48/5-2 (SAE), BO4489/1-1 (FB), BO4489/1-2 (FB), BO4489/3-1 (FB) (www.for2240.de); CRC829 project ID 73111208 (SAE), CRC1403 project ID 1403–414786233 (SAE), FOR2599 project ID 3927 49992 (SAE), Germany’s Excellence Strategy – CECAD, EXC 2030 - 390661388 (SAE). Center for Molecular Medicine Cologne (SAE, DH, CC). EU COST CA18116 (CC; www.aniridia-net.eu).

## Conflict of Interest

The authors declare that the research was conducted in the absence of any commercial or financial relationships that could be construed as a potential conflict of interest.
